# Five years’ experience of an annual course on implementation science: an evaluation among course participants

**DOI:** 10.1186/s13012-017-0618-4

**Published:** 2017-08-02

**Authors:** Siw Carlfjord, Kerstin Roback, Per Nilsen

**Affiliations:** 1 Department of Medical and Health Sciences, Division of Community Medicine, Linköping University, Linköping, Sweden; 2 Department of Medical and Health Sciences, Division of Health Care Analysis, Linköping University, Linköping, Sweden

**Keywords:** Implementation science, Course, Training, Evaluation, Theory, Problem-based learning

## Abstract

**Background:**

Increasing interest in implementation science has generated a demand for education and training opportunities for researchers and practitioners in the field. However, few implementation science courses have been described or evaluated in the scientific literature. The aim of the present study was to provide a short- and long-term evaluation of the implementation training at Linköping University, Sweden.

**Methods:**

Two data collections were carried out. In connection with the final seminar, a course evaluation form, including six items on satisfaction and suggestions for improvement, was distributed to the course participants, a total of 101 students from 2011 to 2015 (data collection 1), response rate 72%. A questionnaire including six items was distributed by e-mail to the same students in autumn 2016 (data collection 2), response rate 63%. Data from the two data collections were presented descriptively and analysed using the Kirkpatrick model consisting of four levels: reaction, learning, behaviour and results.

**Results:**

The students were very positive immediately after course participation, rating high on overall perception of the course and the contents (reaction). The students also rated high on achievement of the course objectives and considered their knowledge in implementation science to be very good and to a high degree due to course participation (learning). Knowledge gained from the course was viewed to be useful (behaviour) and was applied to a considerable extent in research projects and work apart from research activities (results).

**Conclusions:**

The evaluation of the doctoral-level implementation science course provided by Linköping University showed favourable results, both in the short and long term. The adapted version of the Kirkpatrick model was useful because it provided a structure for evaluation of the short- and long-term learning outcomes.

**Electronic supplementary material:**

The online version of this article (doi:10.1186/s13012-017-0618-4) contains supplementary material, which is available to authorized users.

## Background

Implementation science is a fast-growing research field that has emerged in the wake of the evidence-based movement [1–3]. The interest in implementation science has generated a demand for education and training opportunities for researchers in the field and practitioners who are engaged in implementation endeavours. Courses, mainly programs for faculty-level fellows, have been developed and provided by a number of universities in recent years, e.g. by the Implementation Research Institute (IRI) at Washington University in the USA, the National Institutes of Health and Veteran Health Administration in the USA, Trinity College in Ireland, and Radboud University in Nijmegen, Netherlands. A comprehensive description of existing dissemination and implementation (D&I) research training programs can be found in an article by Chambers et al. [4], which discusses variations in formats and other characteristics. The authors state that an in-depth analysis of the quality of programs would aid in the planning of other programs in the area. Assessment of a master-level implementation science course in Germany found that stakeholders’ expectations primarily concerned acquiring knowledge about implementation strategies and knowledge of barriers and enablers, suggesting that implementation *practice* was considered more important than implementation *science* from a stakeholder perspective [5]. It is worth mentioning that education regarding implementation research in health care is provided also by the World Health Organization [6].

In Sweden, a research program called Implementation and Learning (I&L) was launched in 2009 by Linköping University in cooperation with the County Council of Östergötland. The county council (which is responsible for providing health care to approximately 450,000 inhabitants) had identified a need for improved implementation knowledge to facilitate more structured implementation and increased use of evidence-based practices in routine health care practice. The county council’s research and development department took the decision to fund a university research program, which included recruitment of doctoral students with implementation projects and a course in implementation theory and practice. To develop a course curriculum, a group of researchers within the Department of Medical and Health Sciences at Linköping University undertook literature studies and drew on personal experiences of projects involving implementation aspects. Researchers and teachers were recruited from suitable areas of the department, including the Division of Community Medicine and Division of Health Care Analysis, where implementation projects had been conducted in the past. The first course on implementation theory and practice provided by I&L was held in 2011. Since then, the course has been given annually. The demand for the course has been increasing over time, with students coming not only from across Sweden but also from other European countries.

When Proctor et al. [7] evaluated a course provided by IRI at Washington University, St Louis, they concluded that the IRI had been successful in preparing new researchers in the field of implementation science. Meissner et al. [8] evaluated a 1-week course at post-doctoral level provided by the National Institutes of Health and Veteran Health Administration. At the conclusion of the week, the course was positively assessed by the trainees in terms of relevance to their needs/interests, appropriate teaching strategies and gain in confidence in ability to apply the skills and knowledge gained. A university course designed to expand training opportunities in D&I science for public health students and academic researchers, provided by the University of Alabama in 2012–2013, was evaluated by Norton [9]. The course was found effective for simultaneously teaching students and academic researchers about D&I science. However, many of the implementation science courses described or evaluated in the scientific literature are short courses, with limited opportunities to convey a deeper knowledge in the area.

The paucity of implementation science course evaluations has led to a call by Straus et al. [10] in *Implementation Science* for further studies that describe and analyse implementation training initiatives such as graduate curricula in implementation science or continuing professional development courses in implementation. Heeding this call, we undertook an evaluation of the course provided at Linköping University. Hence, the aim of the present study was to provide a short- and long-term evaluation of the implementation science course at Linköping University, applying a widely used training evaluation model developed by Kirkpatrick and Kirkpatrick [11] to investigate the outcome at four levels. The choice of the Kirkpatrick model was based on previous experience of the framework [12].

## Methods

### Design

The study was an evaluation of participation in the specific course provided by the Department of Medical and Health Sciences at Linköping University, Sweden. Two data collections were conducted, one immediately after completion of the course each year and the other as a cross-sectional survey of all former participants in the autumn 2016, i.e. 1–5 years after course participation. The course was evaluated using the model by Kirkpatrick and Kirkpatrick [11], which describes four dimensions of learning from training and education.

### Course curriculum

The course is based on a few key principles. There is an emphasis on the theories, models and frameworks of implementation science to provide a structure and promote an understanding of the mechanisms of implementation for the students. The course does not advocate the use of any specific theoretical approach, instead allowing the students to choose from the existing “smorgasbord” of approaches and reflect on the choices they make. The course applies a systems perspective on implementation, which means that implementation problems and “solutions” are sought at different levels, from individual practitioners to teams, departments, professions, organizations and society. The course advocates interprofessional and interdisciplinary collaboration to facilitate different perspectives on implementation challenges.

The pedagogical method problem-based learning (PBL), widely used at Linköping University, informed the development of the course curriculum [13]. The teachers hold lectures on key topics within implementation science, but consistent with the PBL approach, there are also seminars supervised by a teacher where the focus is on the students’ own discussions of specific themes, such as the meaning of context, the history of the evidence-based movement and implementation outcomes. Further, a great deal of self-study of the literature is expected from the students, in accordance with the PBL approach. Suggestions for literature are provided and further reading is encouraged, but no literature is compulsory. This student-centred approach is sometimes referred to as active learning, characteristics of which includes involvement and engagement in activities, emphasis on developing student skills and exploration of attitudes and values and involvement in higher order thinking in terms of analysis, synthesis and evaluation [14].

The course language is English. The main learning objective of the course is to achieve improved understanding of implementation challenges in health care and increased knowledge concerning relevant theoretical approaches (theories, models, frameworks) used in implementation science.

The course involves three on-site sessions from September to December, over 6 days, including the final 1-day seminar. Few on-site sessions make the course feasible for students from other parts of the country and from abroad. The number of participants has ranged from 20 to 25 over the years. At the start, 25 students were accepted, but this number was lowered to 20 students for practical reasons (availability of rooms, work load for teachers, group sizes). The number of students is considered appropriate to engage the students in discussions as part of the lectures, to avoid teacher-centred approaches and achieve more active, student-centred learning [14, 15]. The class is divided into two groups for the seminars to facilitate active participation by all students. A web-based version of the course was given in spring 2014. It included two on-site occasions, one at the outset of the course and the other being the final seminar. All other lectures, seminars and group discussions were held on-line.

The examination consists of a written assignment, an essay that focuses on the application of a suitable theory, model or framework to a chosen case, e.g. the doctoral student’s own research project. The case consists of either a planned implementation endeavour, which is analysed in terms of potential or actual barriers and facilitators of the process, or the analysis of an already accomplished implementation endeavour. The purpose is to select, motivate and apply a relevant theoretical approach for improved understanding of what might affect implementation success. The final seminar focuses on discussions of the essays, with the authors presenting and defending their essays and other students acting as discussants.

The course has been given each autumn from 2011 to 2016. Some modifications have been made over the years for practical reasons and on the basis of suggestions from the students. An important development is that the course today has less focus on the emergence of implementation science and more focus on modern theories and frameworks used to study implementation. The scope has also been slightly narrowed over time, focusing more on implementation of evidence-based practices in health care settings. The course rendered 7.5 credits in 2011–2012, but this was changed to 5.0 credits from 2013, which was an adaptation to the Linköping University standard for courses at doctoral level. Credits are based on the European Credit Transfer and Accumulation System (ECTS). Approximately eight lecturers, from PhDs to professors, are teaching in the course. The course consists of approximately 25 h of lectures, 10 h of group discussions and seminars and 50 h are expected to be used for literature study and writing of the assignment. The lectures and the themes for the group discussions/seminars in the current curriculum are displayed in Tables 1 and 2.

**Table 1:** Lectures in current curriculum

– Introduction to Implementation Science	
– Theoretical approaches to implementation	
– Strategies to facilitate implementation	
– Research use in clinical practice	
– Individual and contextual influences on implementation	
– Implementation outcomes	
– The role of habit in implementation	
– Innovation research	
– Guidelines in theory and practice

**Table 2:** Themes for supervised group discussions/seminars in the current curriculum

– Defining the implementation object	
– Evidence-based medicine	
– Contextual factors in implementation research	
– Discussion of the short drafts preceding the written assignments	
– Discussion of the written assignments (examination seminar)	

### Framework for evaluation

The Kirkpatrick model [11] was applied as a framework for the evaluation of the course. The model was developed by Don Kirkpatrick in the 1950s and has been widely applied to evaluate training and education in many different settings [16–20]. The model describes four learning outcomes, referred to as levels or dimensions, from the learners’ perceptions immediately after participation in a training initiative to longer term effects in terms of the usefulness of the training. The application of the model in this study is shown in Table 3.

**Table 3:** The Kirkpatrick model for evaluation of education, application in the study

Level	Dimension	Original description and characteristics	Application in this study	Data collection number
1	Reaction	How the participants felt about the training or learning experience	Overall perception of the course	1
2	Learning	Measurement of the increase in participants’ knowledge, before and after	Assessment of learning, knowledge and achievement of course objectives	1/2
3	Behaviour	The extent of applied learning back on the job by the participants	Use of the knowledge gained in the course in general	2
4	Results	Effect on the business or environment by the participants	Use of the knowledge gained in the course in research	2

### Data collection

The first data collection was carried out immediately after completion of the course each year, using an evaluation form distributed face-to-face by one of the teachers and answered and returned anonymously in connection with the final seminar. Students judged the quality of the course on a 5-point Likert-type scale: the extent to which they found the contents useful, the extent to which course objectives were achieved and their overall perception of the course. There were also open-ended questions regarding what was perceived as positive, what was perceived as negative and suggestions for improvement (see Additional file 1). The development of the four questions in the questionnaire was based on discussions among the teachers and was informed by regularly collected information in course assessments at Linköping University. Data are available from all six courses.

The second data collection, which was conducted in autumn 2016, consisted of a questionnaire developed specifically for the present study. Questions were formulated to address the levels 2–4 of the Kirkpatrick model. The questions were discussed among the three authors (who also teach the course) to obtain satisfactory face validity. The questionnaire was pilot-tested with former doctoral students (*n* = 3) interested in implementation, but who had not participated in the course. Minor changes, primarily clarifications, were made based on their suggestions. The eight multiple-choice questions included in the final questionnaire are provided in the “Results” section. There was also one open-ended question regarding what the students believed were the most valuable insights from course participation (see Additional file 2).

The questionnaire was distributed by e-mail to all course participants who had completed the course, using the web-based tool Publech® Survey. Mailing addresses were sought in the course archives. If the e-mail address was not working, an individual search was made to identify the student in order to have an accurate mailing list for the survey. A reminder was sent after 2 weeks, followed by a second reminder a week later.

### Data analysis

Descriptive data from the Likert-type questions in data collection 1 are presented. The five-point Likert scales were transformed into numbers 1–5 and a mean for each of the three questions each year was calculated by hand.

The open-ended questions in data collection 1 concerned factors (i.e. aspects of the course) perceived as positive, factors perceived as negative and suggestions for improvement and were analysed using the basic components of qualitative content analysis described by Graneheim and Lundman [21]. The analysis was initially performed by SC and then discussed among all the authors. Factors perceived as positive are presented separately, whereas factors perceived as negative are incorporated in suggestions for improvement. Data from data collection 2 were handled using Statistical Package for the Social Sciences (SPSS) version 23.0. and are presented descriptively.

## Results

Table 4 shows the number of participants in each course and response rates for the two data collections. The questionnaire in data collection 2 was sent to 101 students. Two e-mail addresses did not work, which meant that 99 students received the questionnaire.

**Table 4:** Course data, number of participants and response rates

Course date	Autumn 2011	Autumn 2012	Autumn 2013	Spring 2014 (Web)	Autumn 2014	Autumn 2015	Total
Credits	7.5	7.5	5	5	5	5	
Students completing the course	24	17	18	5	20	17	101
Responses data collection 1	11	14	16	4	14	14	73
Response rate data collection 1	46%	82%	89%	80%	70%	82%	72%
Responses data collection 2^a^	13/23	8/17	12/18	17/24^b^		12/17	62/99
Response rate data collection 2	57%	47%	67%	71%		71%	63%

^a^The number of questionnaires distributed has been adjusted according to functioning e-mail addresses

^b^In data collection 2, participants in the spring and autumn courses cannot be separated

Of the respondents in data collection 2, 82% were doctoral students when they attended the course. Their current work consisted mainly of research (64%) or health care development (16%). Health care development work includes, for example, work with various quality improvement projects and guideline implementation, which are common tasks for nurses and allied health professionals holding a doctoral degree.

The open-ended question in data collection 2, concerning the most important insights or experiences gained from the course, was answered by 42 respondents. The results are presented according to the Kirkpatrick model.

### Reaction

Reaction was defined as the overall perception of the course based on data from data collection 1. The response options ranged from very negative (1) to very positive (5). The mean value for the 5 years varied from 4.1 to 4.8 (median, 4.6). The extent to which the students found the content useful was rated on a scale from “not at all” (1) to “to a very high degree” (5). The mean value for the 5 years varied from 4.3 to 4.7 (median, 4.5).

The analysis of the open-ended questions from data collection 1 revealed that the positive comments could be attributed to the three categories, Content, Resources and Structure. Regarding Content, the students stated that the course provided a very good overview of implementation research, covering a breadth of relevant topics in the field. The lectures and seminars were perceived to be of high quality, and the emphasis on theories and their application was particularly appreciated. With regard to Resources, the students found the lecturers to be knowledgeable and the literature relevant. The possibility of networking with other doctoral students engaged in implementation science projects was valued. For Structure, the assignment in the form of a written essay was highly appreciated, as were the group discussions. The students also valued the opportunity to apply knowledge gained in the course to their own doctoral projects. The combination of on-site sessions and individual study was also mentioned as a favourable aspect of the course.

Suggestions for improvement in the first years of the course primarily concerned content and structure. The students requested a stronger emphasis on theory; they called for more group discussions, more in-depth lectures and higher requirements for the written assignment. In the later courses, when the course content and structure had been adjusted based on the previous course evaluations, students suggested more emphasis on policy implementation and implementation outcomes.

### Learning

Learning was defined as the students’ assessment of learning, knowledge and achievement of course objectives, based on data collections 1 and 2. A detailed description of the answers from data collection 2 can be found in Table 5. Data collection 1 assessed the extent to which the students believed the course objectives were achieved, rated from “not at all” (1) to “to a very high degree” (5). The mean value for the courses varied from 4.1 to 4.8 (median, 4.5).

**Table 5:** Knowledge in implementation and experiences from the course, results from data collection 2

Question	I consider my current knowledge in implementation to be…	The course has contributed to my current knowledge in implementation issues to a…	I have had use for knowledge gained from the course to a…	In my research, knowledge gained from the course has been valuable to a…	In my work (aside from research), knowledge gained from the course has been valuable to a…
	*n* (%)	*n* (%)	*n* (%)	*n* (%)	*n* (%)
Excellent	5 (8.1)				
Very good	16 (25.8)				
Good	35 (56.5)				
Fair	6 (9.7)				
Poor	0				
Very large extent		21 (33.9)	11 (17.7)	12 (19.7)	6 (13.1)
Large extent		28 (45.2)	30 (48.4)	24 (39.3)	25 (24.1)
Moderate extent		12 (19.4)	18 (29.0)	18 (29.5)	23 (40.7)
Small extent		1 (1.6)	3 (4.8)	7 (11.5)	7 (17.1)
Very small extent		0	0	0	0

In data collection 2, 34% of the respondents considered their knowledge in implementation to be “very good” or “excellent”, while another 56% believed it was “good”. Of the respondents, 79% stated that the course contributed to their current knowledge in implementation to a large or very large extent.

### Behaviour

Behaviour was defined as the self-reported use of the knowledge gained in the course. Two-thirds (66%) of the respondents stated that they have had use for the knowledge to a large or very large extent (Table 5). The open-ended question from data collection 2 revealed that one of the most valuable outcomes of the course was that the students obtained an overview and different perspectives of implementation science. Among the insights gained in the course, the students mentioned improved understanding of the complexity and challenges of implementation endeavours and the importance of planning and structure to succeed with implementation.

### Results

Results was defined as the self-reported use of the knowledge gained in the course in research projects and/or work apart from research activities. The knowledge acquired was reported to have been slightly more valuable for use in research than in other work, as shown in Table 5. The open-ended question showed that the students found the different theories, models and frameworks presented in the course applicable to their own research projects. They also appreciated the research networks that became available to them through course participation.

## Discussion

This study presents an evaluation of an implementation science course provided annually at Linköping University since 2011, applying the model described by Kirkpatrick and Kirkpatrick [11]. The Kirkpatrick model was found to be useful for investigating the results, with the four levels (reaction, learning, behaviour and results) providing a structure for the short- and long-term evaluation of the learning outcomes.

The results showed that the students were very positive after course participation, with the respondents rating high on overall perception of the course and the content (reaction). The respondents also rated high on achievement of the course objectives and considered their knowledge in implementation science to be very good, something that they largely attributed to the course participation (learning). Knowledge gained from the course was viewed to be useful (behaviour) and was applied to a considerable extent in research projects and other work (results).

There are several possible explanations for the favourable results seen in the evaluation. It is obvious that the students are highly motivated to attend the course to learn more about the aspects of implementation science of relevance to their own doctoral projects. The students are highly “self-selected”, suggesting that their motivation is autonomous, i.e. reflecting personal interests and values, according to the Self-Determination Theory [22]. A considerable body of research exists that shows that more autonomously motivated behaviours are more stable, performed with greater care and quality and accompanied by more positive experiences [23].

Implementation science is a relatively new and an evolving field, which means that many doctoral students do not have supervisors or co-supervisors who have research experience in this field. Meeting the course lecturers and other doctoral students thus provides an important opportunity to learn more. The results of the evaluation showed that the students perceived the lecturers to be knowledgeable. Meeting and discussing with likeminded doctoral students also seem to be appreciated; the findings of the evaluation suggest that networking with other students is very important. The Cognitive Evaluation Theory, which is a sub-theory of the Self-Determination Theory, posits that autonomous motivation is facilitated by a sense of relatedness, which is the extent to which individuals perceive that they have a shared experience and meaningful relationships with others [22, 23].

Another likely success factor is the focus on presenting and discussing theories, models and frameworks, i.e. theoretical approaches, used in implementation science. The evaluation results clearly show that the students appreciate the emphasis on theoretical approaches in the course. A paper by Nilsen [3] was developed specifically to account for student requests for clarification on how various theoretical approaches differ from each other. Most students apply one or more of these approaches in their own doctoral projects, which makes the discussions in the course, e.g. about differences and similarities between different approaches, valuable because they provide a context for improved understanding of different approaches.

The course combines more traditional lectures with discussion seminars, but the lectures also allow for a great deal of discussion. The course is limited to about 20 students (the precise number has differed somewhat over the years), which is consistent with the aim of facilitating activity in discussions by the students. The evaluation results concerning the discussion seminars and the examination task, which are presented and discussed in two parallel seminars, indicate that these are important components of the course. Research has shown that practice change is more likely by means of interactive education than through the use of more passive lectures and similar formats [24].

Reflection is an important aspect of the course. The PBL method, which inspired the course curriculum, is a student-centred method intended to encourage reflection and enhance ongoing learning [25]. The method has been positively evaluated [26, 27] and is widely used at Linköping University. Because the course does not advocate the use of any specific theoretical approach, the students can choose from existing approaches, but in their written assignment, they are required to justify the choice they have made. The concept of reflection is generally understood as a means of translating experience into learning, by examining one’s responses, beliefs and actions, to draw conclusions to enable better choices or actions in the future [28, 29]. We believe that the use of active learning contributed to the students’ favourable rating of short-term learning, but also had a positive impact on the longer term behaviour and results. The knowledge gained from this evaluation will be valuable for further improvement of the course, which should be adapted to the preferences and needs of the students for their future research as well as to the developments in implementation science.

### Limitations

This study has several limitations that must be considered when interpreting the results. The response rates to the two questionnaires were acceptable (between 46 and 89% to data collection 1 and between 47 and 71% to data collection 2). However, there could be some response bias because non-responders in survey research can be quite different from those who participate [30]. The total number of course participants was limited and based on the low number of participants, we decided not to ask for demographic data to avoid the risk of individuals being identified. The low number and lack of demographic data meant that we did not perform subgroup analyses or analysis of non-responders. By including students from five consecutive years, we had a total of 101 students. However, this also means that considerable time has passed from course participation to follow-up for those who participated in the early years of the course (range, 1–5 years). The lowest response rates were found in the earlier years, which could have influenced the results, because course development and improvement over time may have led to higher satisfaction among the students. The use of self-reported data must be considered a limitation, as it is often associated with social desirability, i.e. the tendency to answer questions in a manner that will be viewed favourably by others [31]. In our case, the fact that data collection 1 was performed face-to-face may also have influenced the results.

The questionnaire for data collection 2 was pilot-tested by people who did not participate in the course, as we did not want to further limit our population. These individuals, however, were selected among people with relevant knowledge in the area. Still, it might have influenced the development of the questionnaire if they had had personal experience from the course. Not asking for longer term indicators of academic (e.g. publications and research grants) or practice (e.g. developing new intervention approaches) could also be considered a limitation. A competence classification of the PhD students seeking additional implementation science training through our course would have been useful when evaluating the learning progression. Since the long-term evaluation was not planned from the beginning, this information was not obtained. According to the classification in Padek et al. [32], a majority would be classified as beginners.

## Conclusions

The doctoral-level implementation science course provided by Linköping University since 2011 was evaluated with favourable results. The course participants who responded to the questionnaires had a positive view of the professional value of the course, from both short- and long-term perspectives. The overall perception of the course was rated highly, as were the content and achievement of the course objectives. The respondents’ gain in implementation science knowledge was largely attributed to participation in the course, and this knowledge was considered to have a significant value in research projects and other professional activities. The pedagogical approach of active learning seems to have contributed to the students’ positive attitudes and experiences. The adapted Kirkpatrick model was useful because it provided a structure for the evaluation of short- and long-term learning outcomes.

## Additional files

Additional file 1: Questionnaire data collection 1. (DOCX 14 kb)

Additional file 2: Questionnaire data collection 2. (DOCX 14 kb)
