# Supplementary material for: Five years’ experience of an annual course on implementation science: an evaluation among course participants
Source: Implement Sci. 2017 Aug 2;12:101. doi: 10.1186/s13012-017-0618-4 (PMC5541724; doi:10.1186/s13012-017-0618-4)
Supplement: Supplementary file 1 — Questionnaire data collection 1. (DOCX 14 kb) [file 13012_2017_618_MOESM1_ESM.docx]

***Course evaluation***

*Implementation –Theory and Application in Health Care*

**1 – What is your overall perception of the course?**

🞏 Very positive
🞏 Quite positive
🞏 Neither positive or negative
🞏 Quite negative
🞏 Very negative

**2 – To what extent has the course been useful for your learning in implementation science?**

🞏 To a very large extent
🞏 To a quite large extent
🞏 To some extent
🞏 To a small extent
🞏 Not at all

**3 – To what extent did the course content contribute to your achievement of the learning outcomes?**

🞏 To a very large extent
🞏 To a quite large extent
🞏 To some extent
🞏 To a small extent
🞏 Not at all

**Open-ended questions:**

**What do you consider the most positive aspects of the course?**

**What do you consider least positive aspects of the course?**

**What changes would you suggest when the course is given the next time?**
