# Supplementary material for: Five years’ experience of an annual course on implementation science: an evaluation among course participants
Source: Implement Sci. 2017 Aug 2;12:101. doi: 10.1186/s13012-017-0618-4 (PMC5541724; doi:10.1186/s13012-017-0618-4)
Supplement: Supplementary file 2 — Questionnaire data collection 2. (DOCX 14 kb) [file 13012_2017_618_MOESM2_ESM.docx]

To participants in the PhD course ”Implementation – Theory and Application in Health Care” at IMH, Linköping University during the time period 2011-2015

1. I attended the course in the year…

🞏 2011 🞏 2012 🞏 2013 🞏 2014 🞏 2015

2. I was a PhD student at the time when I attended the course

🞏 Yes
🞏 No – if no, which was your work title? ____________________________________

3. My current work consists mainly of…

🞏 Research
🞏 Healthcare development
🞏 Teaching
🞏 Other – please specify _______________________________________________

4. I consider my knowledge about implementation theory (frameworks, models and concepts) to be

🞏 Excellent
🞏 Very good
🞏 Good
🞏 Fair
🞏 Poor

Comments: ______________________________________________________________________

5. The course has contributed to my current knowledge about implementation issues to a

🞏 Very large extent
🞏 Large extent
🞏 Moderate extent
🞏 Small extent
🞏 Very small extent

Comments: ______________________________________________________________________

6. I have /have had use for knowledge gained from the course to a

🞏 Very large extent
🞏 Large extent
🞏 Moderate extent
🞏 Small extent
🞏 Very small extent

Comments: ______________________________________________________________________

7. In my research, knowledge gained from the course has been valuable to a

🞏 Very large extent
🞏 Large extent
🞏 Moderate extent
🞏 Small extent
🞏 Very small extent

🞏 Not relevant

Comments: ______________________________________________________________________

8. In my work (aside from research), knowledge gained from the course has been valuable to a

🞏 Very large extent
🞏 Large extent
🞏 Moderate extent
🞏 Small extent
🞏 Very small extent

🞏 Not relevant

Comments: ______________________________________________________________________

9. What do you consider the most important insights or experiences that you gained from the course?

______________________________________________________________________

Thank you for your participation!
